# Near-falls in Singapore community-dwelling older adults: a feasibility study

**DOI:** 10.1186/s40814-020-00748-1

**Published:** 2021-01-12

**Authors:** Shawn Leng-Hsien Soh, Chee-Wee Tan, Judith Lane, Ting-Ting Yeh, Benjamin Soon

**Affiliations:** 1 Singapore Institute of Technology, 10 Dover Road, Singapore, 138683 Singapore; 2 Glasgow Caledonian University, Cowcaddens Rd, Glasgow, G4 0BA UK; 3 Queen Margaret University, Queen Margaret University Way, Musselburgh, EH21 6UU UK

**Keywords:** Near-falls, Geriatrics, Community-dwelling, Older adults, Balance recovery

## Abstract

**Background:**

A near-fall is defined as a loss of balance that would result in a fall if sufficient balance recovery manoeuvres are not executed. Compared to falls, near-falls and its associated balance recovery manoeuvres have been understudied. Older adults may not recognise a near-fall or identify the use of their balance recovery manoeuvres to prevent a fall. The consensus on the methods to collect near-fall data is lacking. The primary objective of this study was to determine the feasibility of recruitment and retention. Secondary objectives were to establish evidence that Singapore community-dwelling older adults can identify near-falls and associated balance recovery manoeuvres. Texting and calling methods were explored as reporting methods.

**Methods:**

This study took place in Singapore (September to October 2019). Participants were healthy, community-dwelling adults aged 65 or older. Recruitment was done through poster advertisement, and all participants gave informed consent. Participants attended a briefing session and reported their near-fall or fall incidence over 21 days using either daily texting or calling. The primary outcome measures were the recruitment rate, retention rate, preferred modes for data reporting and ability to report near-falls or falls. Secondary outcomes included the self-reported incidence of falls and near-falls.

**Results:**

Thirty older adults were recruited in 5 weeks. All participants completed the study. They understood near-fall concepts and were able to report the occurrence and relevant balance recovery manoeuvres used to prevent a fall. 87% (26/30) chose to text while 13% (4/30) selected calling as their reporting method. One actual fall (0.16%) out of 630 responses was reported. Thirty-six incidents (5.7%) of near-falls were recorded. Sixteen participants (53.3%) experienced near-falls and half of this group experienced two or more near-falls. The use of reach-to-grasp strategy (36%), compensatory stepping (52.8%), and other body regions (11.2%) were used to prevent the fall.

**Conclusions:**

The study provided evidence that studying near-falls in Singapore community-dwelling older adults is feasible and can be applied to a large-scale study. Recruitment and retention rates were good. Older adults were able to identify near-falls and balance recovery manoeuvres. Both texting and calling were feasible reporting methods, but texting was preferred.

**Trial registration:**

ClinicalTrials identifier: NCT04087551. Registered on September 12, 2019

**Supplementary Information:**

The online version contains supplementary material available at 10.1186/s40814-020-00748-1.

## Key message regarding feasibility

1. What uncertainties existed regarding the feasibility?

- It is unknown whether Singapore community-dwelling older adults could determine near-falls and associated balance recovery manoeuvres.
- There was a lack of an operational definition of near-fall to be used in near-fall studies.
- There is no consensus on the reporting methods to collect near-fall data.

2) What are the key feasibility findings?

- Singapore community-dwelling older adults were able to identify near-falls, and various balance recovery manoeuvres used to prevent falls such as compensatory stepping and reach-to-grasp strategies.
- The operational definition of near-fall and the reporting methods used in the study were well adopted by the Singapore community-dwelling older adults.
- There were good recruitment and retention of Singapore community-dwelling older adults in the study to investigate near-falls.

3) What are the implications of the feasibility findings for the design of the main study?

- Singapore community-dwelling older adults were able to relate to the near-fall concepts, supporting that a large-scale study can be conducted to study near-falls.
- The feasibility of the operational definition of near-falls and data collection methods provided evidence to justify methods in the main study.
- The findings on the recruitment rate and retention rate may be used to justify a reasonable sample size to be calculated for a large-scale study.

## Background

Falls in older people can lead to devastating health and social consequences, including serious injuries, hospitalisation, loss of independence, diminished quality of life and depression [1, 2]. Global estimates of the burden of falls have remained substantial [3], i.e. falls have been ranked as the 18th leading cause of the age-standardised rate of disability-adjusted life years [4] and have been identified to be the second leading cause of death due to unintentional injuries [5]. Recent studies have begun recommending that clinicians should pay more considerable attention to near-falls [6, 7]. Near-falls relate as a loss of balance that would result in a fall if sufficient balance recovery mechanisms are not activated [8]. These mechanisms, which include the postural adjustments (“fixed-support” strategies) and use of upper and lower limbs (“change-in-support strategies”), are critical recovery strategies preventing a fall caused by perturbations, e.g. slips, trips and missteps, collisions or other interactions with the environment and destabilising effects of volitional movement [8, 9].

Near-falls in older people are common and can be a risk factor for falls. Near-falls occur more frequently compared to an actual fall [6]. More than half of the community-dwelling older adults have experience occasional or frequent near-falls [10, 11], and a third have reported a near-fall at least once a month [6, 11]. The experience of near-falls among older adults has been shown to be an independent predictor of a subsequent fall irrespective of the physical frailty in community-dwelling older adults [12]. Despite the high incidence and predictive nature of near-falls, there are very few studies examining related issues. One reason is that adults often do not recognise or attach any significance to the transient event [13].

While everyone tacitly knows what a near-fall is, a concrete definition of a near-fall has been lacking. A vague understanding can create ‘faulty transmission of information between patient and physician and between researchers.’ [14]. However, it has been challenging to operationally define near-fall comprehensively for laypeople [15]. The existing interpretations have been unspecific about the balance recovery manoeuvres used to arrest a fall. Some of these interpretations have included ‘a loss of balance regained before striking the ground’ [13], ‘events where subject almost falls but is able to catch him/herself or to stop the fall’ [16] or ‘misstep relating to a trip, slip or other loss of balance in which recovery occurred to prevent a fall’ [17]. Maidan and colleagues [8] detailed various recovery mechanisms suggesting that at least two of the following compensatory mechanisms should be activated to determine the event as a near-fall: (i) unplanned movement of arms or/and legs, (ii) unplanned change in stride length, (iii) lowering of the centre of mass, (iv) unplanned change in stride velocity and (v) trunk tilt. These descriptions, with the elaborated recovery strategies, could pose a challenge for older people to comprehend kinesiology jargons. A relevant and comprehensive definition of a near-fall easily understood by an older person is needed.

Various documentation methods to study falls such as questionnaires, fall diaries or telephone calls had been used widely [12]. However, these methods had not been studied much with collecting near-fall data. One notable concern is the accuracy of the data reported. Some older people have expressed recall difficulty [18] with issues of under-reporting and over-reporting which may create erroneous data collection [15]. The errors reported by previous studies have included forgetting to record a fall in the falls diary or reporting a salient event into that particular period which occurred outside of the recall period (i.e. ‘telescoping’) [19]. In order to reduce these errors, Ryan and colleagues [20] applied a data collection method of using a daily telephone call (calling) at a prearranged time to improve data accuracy. None of the study participants had reported any difficulty recalling a fall or near-fall event daily over the 3-week study period. The authors reported a high compliance rate of 96.7%. While calling had been a useful method to collect near-fall data, this method was applied three decades ago and should be evaluated in today’s context when many people have started becoming active mobile phone users. A census study conducted in Singapore reported 98% of households own at least one mobile phone [21]. 95% of the Singapore population using the mobile phone to browse the internet [22]. Today, texting is a common mode of communication. A comparison between calling and texting as a preferred method among community-dwelling older adults to collect near-falls or falls data needs further investigation.

Singapore, being one of the most densely populated countries in the world [23], faces an unprecedented demographic shift towards an aged society. The proportion of the resident population age 65 years and over will significantly increase from 1 in 6 (590,000) in the year 2020, to 1 in 4 (900,000) by the year 2030 [24]. This rapid transition to a hyper-aged society poses significant challenges relating to access, quality, efficacy and funding for the healthcare services. Initiatives to promote ‘successful ageing’ and ‘ageing-in-place’ in the community included creation of a conducive senior-friendly environment for older people to move around safely and confidently within their homes and also within their community [25]. However, the impacts of these efforts on the person-environment interactions concerning near-fall or falls have been understudied. To the best of the authors’ knowledge, there have been no local studies on near-falls in Singapore community-dwelling older adults. The new knowledge gained from this work will provide a deeper understanding of the methods required to study near-falls in older adults. This research aimed to obtain evidence that to study near-falls in Singapore community-dwelling older adults is feasible, and the methods can be applied to a large-scale study.

### Objectives

- To evaluate the recruitment process and retention of community-dwelling older adults in a local study of near-falls.
- To assess the use of a briefing to explain the different definitions between a near-fall and a fall to the community-dwelling older adults.
- To determine the use of daily texting compared to calling as suitable data collection methods for an appropriate trial design relating to (i) participant preference (ii) adherence to protocol.

## Methods

### Study design and ethics

This feasibility study was an observational cohort study. Data protection and ethical approval were obtained from two institutional ethics review boards, Singapore Institute of Technology (reference number: 2019129) and Queen Margaret University (reference number: REP0197). This study conforms with the CONSORT extension to randomised pilot and feasibility trials, excluding specific items required for randomisation nature of the study [26] (Additional file 1).

### Participants and setting

Poster advertisement was circulated to the network of older adults engaged by Singapore Institute of Technology (SIT) for school assignments, residents’ network centres and various clinical partners across Singapore. Between September and October 2019, interested older adults contacted the researcher through the contact details listed in the posters or given through word-of-mouth recommendations. They were asked by a researcher whether they were aged 65 years or older, living in the community and were able to read, write and communicate in English before a meeting was arranged at SIT or an agreed location in Singapore. During the meeting, they were provided study information, e.g. how the study would be conducted, what will be expected of them, the study’s eligibility criteria (Table 1). They were informed that participation would be voluntary. If they did not meet the eligibility criteria, they were given general information about falls prevention. An opportunity to ask questions was offered, and the consent form was completed if they agree to participate. In line with good practice, participants were given SGD$50 as a thank you for taking part, reimbursing them for their time, contribution and any expenses incurred, e.g. sim-card cost, travelling cost. This was not used to induce participation in the study.

**Table 1:** Eligibility criteria

Inclusion criteria	Exclusion criteria
65-year-old and above	Requiring any physical assistance from another person to walk within home
Ability to read, write and communicate in English	Known active malignant conditions
History of at least one near-fall or one fall within the last 12 months	Cardiovascular conditions, e.g. neurally mediated syncope, cardiac syncope, structural heart diseases, e.g. aortic stenosis or hospitalization for myocardial infarction or heart surgery within 3 months
Living independently in the community with or without the use of a walking aid	Pulmonary conditions, e.g. chronic severe obstructive pulmonary disease or oxygen dependence
Not having any cognitive dysfunction by achieving a score of 7 or less in the 6-item cognitive impairment test (6CIT) [27]	Musculoskeletal conditions, e.g. moderate to severe osteoarthritis that could affect balance control and muscle function, e.g. self-reported pain or dysfunction of the trunk and extremities, fractures or injuries in the extremities in the last 6 months.
Able to walk 6 m within 12 s by performing the Timed Up and Go (TUG) test [28]	Neurological conditions, e.g. Parkinson’s disease, sequelae of stroke, amyotrophic lateral sclerosis, multiple sclerosis or severe dementia or epilepsy
Able to catch a 30-cm ruler with each hand using the Hand Reaction Time (HRT) test [29]	Legal blindness, severe visual impairment, severe hearing impairment or legal deafness

### Pilot sample size

Based on Ryan et al. [20] study, we estimated an 80% response rate and a 10% drop out rate for our research. We adopted a sample size of 30, which had been identified to be a reasonable number for a feasibility study [30]. A projected number of 630 responses (30 subjects for 21 days) were to be obtained throughout the research.

### Data collection

The researcher completed the data collection with the participants using a standardised data extraction form to record demographic data (age, race, gender, educational level, housing type, living situation, personal mobility, falls history and near-fall history), cognitive functioning using the Six-item Cognitive Impairment Test (6CIT) [31], upper limb reaction function using the Hand Reaction Time Test (HRT) [29] and lower limb physical function using the Timed Up and Go Test (TUG) [28].

### Cognitive functioning

The 6CIT is a brief and simple validated tool used for cognitive screening in the community-dwelling older adults [27]. Participants needed to complete three tests of temporal orientation (year, month, time), two tests of attention (counting backwards from 20 to 1; reciting the months of the year in reverse) and short-term memory (5-item address). The total score was recorded with higher scores indicating greater impairment.

### Upper limb reaction function

The HRT [29] is a performance measure to determine whether the participant will be able to execute grasping manoeuvre quickly. A 30-cm ruler will be dropped between the participant’s thumb and index finger, with instructions to ‘catch’ the ruler between the fingers as quickly as possible. The participant had to grip the ruler after it is dropped without letting the ruler landing on the floor. The test established whether the participants had adequate upper limb reaction ability.

### Lower limb physical function

The TUG is a reliable and valid test for quantifying functional mobility in older adults [28]. Participants were timed to complete the task of raising from an armchair, walk 3 m, then walk back at their normal pace to sit down in the armchair in a safe manner. The time taken to complete the task was recorded.

### Key research outcomes

#### Briefing to explain a fall and near-fall

One primary outcome measure was to determine the feasibility of conducting a presentation to explain the different meanings between a near-fall and a fall to the community-dwelling older adults. Operational definitions of falls and near-falls were presented to the participants. These definitions were consistent to those in the literature using language and concepts that aimed to be clear, relevant and easily understandable by the older participants.

A fall definition was adopted, in concordance with the PROFANE-group consensus statement, as ‘an unexpected event in which the participant comes to rest on the ground, floor or lower-level’ [15]. Explaining the concept of falls in a lay perspective to the participants included scenarios involving a slip or a trip or any event causing a loss of balance resulting the individual to land on a lower level including the floor, ground or furniture such as chair or bed. The participants were informed that intentional causes such as a deliberate push by another person or a medical occurrence such as heart attack, fainting, stroke and seizure were not considered as falls in the study.

Near-fall was defined as an event when the individual slips, trips or loses balance but uses the hand(s) or leg(s) or any body part to recover balance and prevent a complete fall. This definition aimed to be relevant, comprehensive and understandable to the older participants. Participants were then presented with several scenarios (Table 2) and asked whether each scenario reflected a fall, a near-fall or no fall. If the participant identified the situation as a near-fall, then the researcher asked what balance recovery manoeuvre was used to prevent the fall. At the end of the briefing, the researcher ensured there were no further questions from the participants about differentiating a fall, near-fall or no fall event.

**Table 2:** Scenarios given to participants

1. Fall scenario – The individual is walking along the street, trips over an object and loses balance. The individual landed on the floor.	
2. Fall scenario – The individual is getting dressed by the bed, loses balance and lands on the bed.	
3. No fall scenario – The individual is walking across the room and starts to feel dizzy. The person sits on a nearby chair.	
4. No fall scenario – The individual is walking along the street and is deliberately pushed by another person to the ground.	
5. Near-fall scenario – The individual holds onto a rail after losing balance when the bus starts to move (hand strategy)	
6. Near-fall scenario – The individual stumbles while walking and can restore balance by taking a few steps (leg strategy)	

#### Collecting near-fall and fall data

After the briefing, the researcher obtained the preference of the participants’ mode of communication to report the incidence of near-fall, fall or no fall. Three options were provided: (1) a daily call or (2) a daily text or (3) either a daily call or text scheduled at a prearranged timing. There were no text reminders given during the day to avoid overburdening the participants. One scheduled text was sent each day, even if there was no text reply from the participant. For calling, a second call would be made an hour later of the scheduled timing if there was no response to the first call. No further calls were made if there was no response to the second call. Over the next 21 days, the participants were asked two questions by the researcher daily, ‘Have you fallen in the past 24 hours?’ and ‘Have you almost fallen in the past 24 hours?’ using the participant’s preferred mode of communication. If ‘yes’ was replied to the near-fall question, the participants were asked, ‘Did you prevent the fall using your hands or legs or any body part?’ The participants then identified the balance recovery manoeuvre used to prevent the fall. If ‘yes’ was replied to the fall question, the researcher checked if the participant was able to continue with the study. A 21-day follow-up duration was selected to replicate the study period applied by Ryan and colleagues [20]. All data were recorded in a logbook by the researcher. No details of the fall or near-fall events were obtained. Participants were informed that they were able to contact the researcher either through text or telephone if needed when it is only safe to do so.

#### Statistical analysis for the pilot study

The feasibility outcomes were summarized descriptively and narratively. Descriptive statistics were used to summarize recruitment, retention, sample characteristics, incidence frequency of near-falls and falls and the types of balance recovery mechanisms used in near-falls.

## Results

### Recruitment and retention

**Fig. 1:**
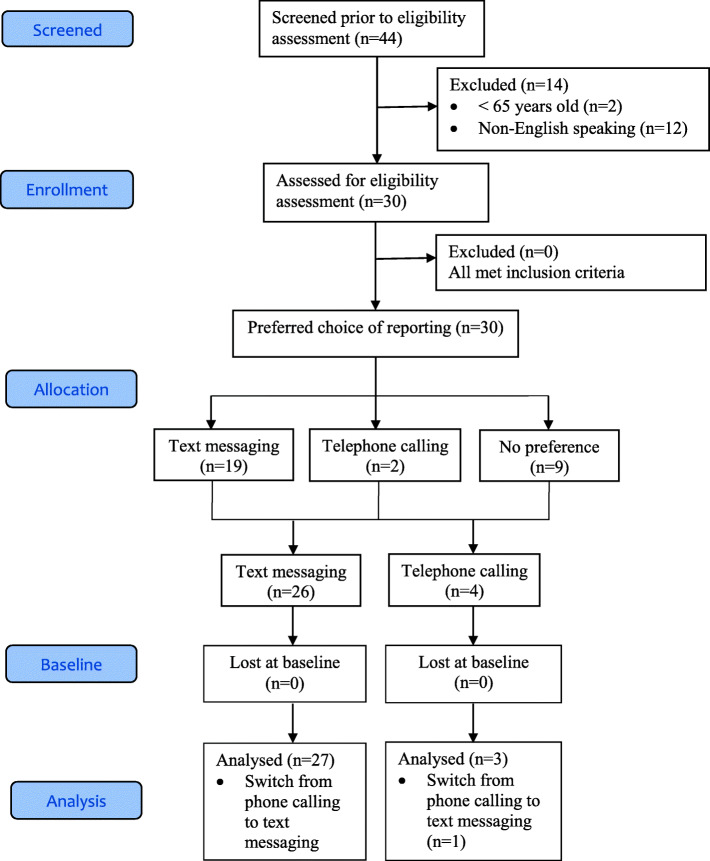
CONSORT 2010 flow diagram for the feasibility study

The recruitment for the study was estimated to be at a rate of three to five participants per week, and the study was projected to complete in 8–12 weeks. The study completed within 8 weeks because of the outreach to the network of older adults formerly recruited for the school’s projects and the strong links that the research team has with the various community groups in Singapore. Of the 44 community-dwelling older adults screened, 30 were eligible for this study. The reasons for the exclusion (*n* = 14) were (a) 2 adults did not meet age criteria and (b) 12 older adults were non-English speaking, e.g. Mandarin. The remaining 30 participants met the eligibility criteria and were enrolled in the study. See Fig. 1 for the CONSORT 2010 flow chart. At the end of the study, no participants dropped out of the study.

### Participants’ characteristics

The participants’ characteristics were presented in Table 3. There were slightly more male (*n* = 16, 53.3%) compared to female (*n* = 14, 46.7%), aged between 65 and 85 years (72 ± 5.2). There was a fair ethnic representation of the Singaporean community with Chinese (*n* = 22, 73.3%), Malay (*n* = 5, 16.7%), Indian (*n* = 2, 6.7%) and Eurasian (*n* = 1, 3.3%). There was mixed participation of individuals with different education level, including primary level (10%), secondary level (53%) and tertiary level (37%). About half of the group lived in a 5-room or executive flat-type property and the others in 3-room flat (16.7%), 4-room flat (13.3%) and private housing estates (23.4%). Most of them were living with others (90%). All were community ambulant with some using a walking stick in the community (10%). Thirteen participants (43.3%) had fallen at least once in the last year, and 10 participants (33.3%) had experienced one or more near-falls in the last month. Nineteen participants (63.3%) reported they have never or rarely experienced a near-fall in the last one year while 11 (36.7%) reported occasional or frequent near-falls.

**Table 3:** Baseline characteristics of participants

Characteristic	***N*** = 30
**Age range (mean)**	65–85 (72)
**Gender**	
Female, *n* (%)	14 (46.7)
**Ethnicity**	
Chinese, *n* (%)	22 (73.3)
Malay, *n* (%)	5 (16.7)
Indian, *n* (%)	2 (6.7)
Eurasian, *n* (%)	1 (3.3)
**Housing type**^**a**^	
3 room apartment, *n* (%)	5 (16.7)
4 room apartment, *n* (%)	4 (13.3)
5 room or executive apartment, *n* (%)	14 (46.7)
Condominium or other apartments, *n* (%)	5 (16.7)
Landed property, *n* (%)	2 (6.7)
**Education level**	
Primary, *n* (%)	3 (10)
Secondary, *n* (%)	16 (53.3)
College/University, *n* (%)	11 (36.7)
**Living situation**	
Alone	3 (10)
With others, *n* (%)	27 (90)
**Personal mobility**	
Independent	27 (90)
Use of walking stick, *n* (%)	3 (10)
**History of falls in the last one year**	
0	17 (56.7)
1 or more	13 (43.3)
**History of near-falls in last one month**	
0	20 (66.7)
1 or more	10 (33.3)
**History of near falls in last one year**	
Never or rarely	19 (63.3)
Experience occasional or frequent	11 (36.7)
**Six-Item Cognitive Impairment Test (points)**	
0–3	26 (87.7)
4–7	4 (13.3)
**Timed up and go test (seconds)**	
< 9.0	14 (46.7)
9.1–10.0	6 (20)
10.1–11.0	6 (20)
11.1–12.0	4 (13.3)
**Able to complete Hand Reaction Time Test**	30 (100)
**Preferred mode of communication**	
Daily telephone calls only	2 (6.7)
Daily text messages only	19 (63.3)
Daily telephone calls &/or text messages only	9 (30)

^a^Housing type—a person’s affordability of different types of housing reflected by the type of property with private condominium or other apartments and landed property being more expensive option compared to public housing, e.g. 3-, 4-, 5-room apartments. There are increasing governmental efforts to have more elderly-friendly and barrier-free environment for both public and private housing [32]

### Choice of data reporting method

Out of the thirty participants, nineteen requested texting, two participants chose to have calling, and the remaining nine had no preference to either texting or calling. For those with no preference, seven participants eventually chose texting because they wanted the convenience to report their data at their availability. Two participants decided to receive a call because they preferred to talk to the researcher and wanted to address any concerns that might arise during the study period. During the study, one participant switched from calling to texting because she reported having difficulty talking due to her gum pain.

Compared to texting, calling was preferred when clarifications were needed. Two participants who chose to text, called the researcher to clarify their near-fall. One participant asked if the event was a near-fall when he almost fell after he unintentionally bumped against another pedestrian while he was walking and using his phone. Another described his experience that he recovered using his body without the use of the hands and legs after losing balance on a chair. The researcher recorded these incidents as near-falls, and the balance recovery manoeuvres were the use of the hand and other body parts, respectively. The study did not record the details of these events. It may be beneficial for future studies should investigate the older peoples’ near-fall experiences.

### Briefing of definitions on falls and near-falls

All participants completed a 30-min one-to-one briefing session using a PowerPoint presentation. The use of short video clips illustrated different near-fall scenarios to aid participants’ understanding of the concept of near-falls. All participants reported no issues in identifying and differentiating between falls and near-falls. All correctly answered the six test scenarios.

### Research data collection

All participants reported their near-fall and fall incidence during the study period. A total of 630 events (i.e. no falls, falls or near-falls) (100%) were recorded across the study period. The data received by the researcher was not reported daily by some participants. Over the 21-day period, five participants text-replied on the second day and one participant text-replied on the fourth day in one occasion. One participant text-replied on the second day across four episodes and one participant varied his text-replies up to 4 days through the study. For calling, all participants needed more than one scheduled daily calls to obtain the data over the study period.

None of the participants reported any difficulty remembering whether they had a fall or near-fall during the study period. The concepts of falls and near-falls were well-understood by all participants. However, clarifications were needed for the ‘other body parts’ balance recovery manoeuvres used to recover from a loss of balance, i.e. ‘using the hip to lean against the wall’ or ‘body jerking up’.

During the 3 weeks, one actual fall was recorded. This yielded a fall incidence of 0.1% (1 in 630 records). Near-falls were reported 36 times or an incidence rate of 5.7%. Among the thirty participants, sixteen participants (53.3%) experienced near-falls, and 50% of them experienced two or more near-falls. A comparison was made between older adults who experienced one or more near-fall (i.e. near-fallers) and those who did not experience a near-fall (i.e. non-near-fallers) during the study (Table 4). The near-fallers had a mean age of 70 years and were 4 years younger than the non-near-fallers (74 years). The balance recovery manoeuvres used to prevent the fall were: reach-to-grasp strategy (36%), compensatory stepping (52.8%) and other body regions, e.g. hip and trunk (11.2%).

**Table 4:** Comparison between near-fallers and non-near-fallers

	Near-fallers	Non-near-fallers
	*N* (%)	*N* (%)
Age range (mean)	65–75 (70)	67–85 (74)
Chinese, *n* (%)	13 (81.3)	9 (64.3)
Malay, *n* (%)	2 (12.5)	3 (21.4)
Indian, *n* (%)	1 (6.3)	1 (7.1)
Eurasian, *n* (%)	0	1 (7.1)
3 room, *n* (%)	0	5 (35.7)
4 room, *n* (%)	3 (18.8)	1 (7.1)
5 room, *n* (%)	8 (50)	6 (42.9)
Condo/landed, *n* (%)	5 (31.3)	2 (14.3)
Primary, *n* (%)	2 (12.5)	1 (7.1)
Secondary, *n* (%)	9 (56.3)	7 (50)
College/university, *n* (%)	5 (31.3)	6 (42.9)
Alone, *n* (%)	1 (6.3)	2 (14.3)
With others, *n* (%)	15 (93.8)	12 (85.7)
Independent, *n* (%)	15 (93.8)	12 (85.7)
Walking stick, *n* (%)	1 (6.3)	2 (14.3)
Previous fall last 12 months, *n* (%)	6 (37.5)	7 (50%)
Previous near-fall last month, *n* (%)	3 (18.8)	7 (50%)
Never or rarely, *n* (%)	11 (68.8)	8 (57.1)
Occasionally or frequently, *n* (%)	5 (31.3)	6 (42.9)
6CIT	1.1 (1.6)	1 (1.6)
TUG	9.2 (1.6)	8.9 (1.8)

## Discussion

### Recruitment process and retention

This study had good participation and retention rate. There was an average of five older people recruited into the study each week, completing the recruitment in 5 weeks. There was no difficulty recruiting older people who were able to communicate in English. The profile of the sample suitably represented the Singapore older people community as a multi-ethnic society. They were able to report their encounters with near-falls (if any) based on their interactions between their regular activities of daily living and the environment. However, we found that some older participants were unable to participate in this study as the ability to communicate English was listed as an eligibility criterion. For a future larger-scale study, the study materials may be translated into different languages, such as Mandarin, Malay and Tamil to allow greater participation of older people. In regard to retention, the high adherence of participants in our study was consistent with the study conducted by Ryan and colleagues [20]. It is postulated that the compliance of the older adults in the study was attributable to altruism and convenience of reporting methods [33]. Many older adults reported that they enjoyed participating in these research studies to stay mentally alert and wanted to keep updated on health-related issues. They also identified the importance of near-falls and relevant balance recovery manoeuvres as useful concepts towards helping themselves and other older people to prevent a fall. They shared that the reporting methods did not intrude their regular lifestyle and found them convenient.

### Briefing and working definitions

A 30-minute briefing session conducted was sufficient to ensure that adequate information of the study provided enough understanding to older people, without causing unnecessary mental fatigue [34]. While these sessions were conducted one-to-one, the researchers were of the view that the presentation could be conducted in a group setting during a larger-scale study. The working definitions provided were comprehensible. Older people had no difficulty in learning the definitions and differentiating a fall or near-fall. They were able to apply the definitions into different hypothetical situations and were able to relate to their personal experience. When encountered with a near-fall situation, the older adult was able to identify the balance recovery manoeuvres used to prevent the fall.

The clarifications requested by two participants about near-falls implied some improvement would be needed for the operational definition of near-falls. In the study, the definition of a near-fall was presented as an event where the individual slips, trips or loses balance but uses the hand(s), (leg)s or any body parts to recovery balance and prevent a complete fall. In future, the different types of events (i.e. the type of perturbations) and various balance recovery manoeuvres (i.e. use of the hand(s), leg(s) or body parts) could be further elaborated with the various causes of disequilibrium and the different ways to recover equilibrium respectively. One suggestion would be to define a near-fall as an event when the individual may experience a fall due to external perturbations, such as a slip, a trip or external forces causing the individual to lose balance, or due to internal perturbations such as the movement of the individual resulting in the individual being destabilised [35]. The other suggestion would be to make the explanation of using various body parts to arrest a fall more explicit for individuals to understand the mechanisms of avoiding the fall [8]. This could be the hand grabbing onto a handrail, the legs taking a few quick steps on the floor, the shoulder or hip leaning against a wall or the trunk moving to correct body stability. While all individuals might not easily resonate with all types of recovery strategies occurring during a near-fall, the explicit illustrations may increase awareness among the older people of the broader context of using various balance recovery manoeuvres to prevent a fall following perturbations [36].

### Methods of collecting near-fall data

Data collection methods, such as telephone calls and diary, had been used in previous studies to record near-falls [6, 20]. The application of using texting in this study to collect near-fall data is novel. The new knowledge obtained from the study is that older people preferred texting over calling. This provided evidence that many older adults were receptive to the use of technology in research. In this study, the older adults selected the use of texting to report near-falls data because of its convenience, i.e. they might be busy with other activities and were not able to pick up the call. They were overall comfortable with the use of this mode of communication as they usually text among family and friends. However, calling might still need to be an option for the study of older people. Three participants who opted to call had mobile phones. They preferred the researcher to call them instead of texting because of their discomfort to text or preferred a more personal way of communication.

The evidence in this feasibility study demonstrated a positive predisposition towards the use of texting as a choice of data reporting in Singapore. Both methods using texting and calling were highly successful for recording near-falls or falls. Both participants and the study team appreciated the flexibility of using texting. While scheduled timings were arranged with the participants, not all participants replied immediately to the text messages or picked up the call. All participants reported their data at their own time. It was noted that calling is a more time-consuming method on the part of the researcher to reach the participant. To reduce overburdening of the researchers, a larger-scale study would need to factor in the necessary resources (i.e. a financial budget to hire research assistants or time taken) to conduct the calling.

Nevertheless, both ways confirmed these methods’ potential use for obtaining data that may be easily forgettable. Based on participants’ feedback and the researchers’ subjective impressions, the frequency using a twice-a-week interval to get falls or near-falls data is practical to implement, able to sustain the cooperation of the participants and easy for participants to retain any fall or near-fall details.

## Conclusion

This study demonstrated that the procedures and data collection processes were feasible. It provided evidence that older people can understand the concept of near-falls and identify relevant balance recovery manoeuvres used to prevent a fall. The working definition for falls and near-falls described in this study is comprehensible and relevant to the older adults. Both texting and calling are feasible reporting methods, but older adults in Singapore preferred texting as the more convenient mode of reporting.

## Supplementary Information

**Additional file 1: Table S5.** CONSORT extension for Pilot and Feasibility Trials Checklist. CONSORT 2010 checklist of information to include when reporting a pilot or feasibility trial. A completed CONSORT extension for Pilot and Feasibility Trials checklist.

## Data Availability

The datasets used and/or analysed during the current study are available from the corresponding author on reasonable request.

## Abbreviations

SIT: Singapore Institute of Technology
6CIT: 6-Item Cognitive Impairment Test
TUG: Timed Up and Go Test
HRT: Hand Reaction Time Test
